# Supplementary material for: Using numerical modeling and simulation to assess the ethical burden in clinical trials and how it relates to the proportion of responders in a trial sample
Source: PLoS One. 2021 Oct 11;16(10):e0258093. doi: 10.1371/journal.pone.0258093 (PMC8504716; doi:10.1371/journal.pone.0258093)
Supplement: Attachment — Submitted filename: Manuscript Review Report- PONE-D-21-01394- May-2021.docx [file pone.0258093.s002.docx]

**Manuscript Review Report**

**Manuscript Number:** PONE-D-21-01394

**Journal:** PLOS ONE

**Title:** Using numerical modelling and simulation to assess the ethical burden in clinical trials and how it relates to the proportion of responders in a trial sample

**Summary:** This manuscript reports on a complex hypothetical study which uses statistical and numerical modelling in an attempt to map out the proportion of ‘responders’ and ‘non responders’ in randomized control trials (RCTs) for comparison of new drugs or treatments, in an attempt to reduce the number of ‘non-informative participants’ or ‘unnecessary’ participants in RCTs. By identifying such non-responders in RCTs, the authors argue that this would help to minimize ‘ethical losses’ characterized for example as “exposure to side-effects, loss of access to standard care, or loss of access to 182 the best available and tailored treatment.”

While he manuscript is original ad attempts to resolve some of the ethical and moral dilemmas associated with the RCT format for investigational drugs or therapy. The manuscript as currently written is not easily comprehensible to the ‘non-expert’ or general reader and will require some modifications to enhance clarity and reduce some identified ambiguities in the current manuscript prior to acceptance for publication.

**Suggested areas for further clarification and improvement of the manuscript:**

1. a). In the Abstract the authors appear to use the following terms interchangeably i.e., “non-responders,”; “unnecessary participants” and “non-informative participants” …. The question that comes to mind is this…. Do these terms refer to the same fragment of the sample population, or are they different in anyway?

b). Again, on Page 8, Lines 176-177, the authors state “non-responders, non-informative and unnecessary participants, totaling (sp) the number of non-responders and non-informative participants.”

c). Furthermore, on Page 8, Lines 168-171, the authors state “Elaborating on this same reasoning, the non-responders are unnecessary participants for they do not bring into the trial a part of the experimental treatment efficacy and they cannot (when they were allocated to the control arm) or do not (experimental treatment arm) benefit from the treatment.”

One would like to suggest that this apparent use of these different terminologies of ‘non-responder’, ‘unnecessary participants’ and ‘non-informative participants’ creates a confusion for the reader who has to try to decipher the meaning of each of this categories…I therefore suggest that the authors should clearly define each of these categories…Are they the same or are they different?...If they are the same then say so…if they are different , then clearly define how they are different from each other? Are some of these categories essential to the RCT format…if so, which ones are essential or non-essential?... The way to approach this is suggested in 2. Below:

2. On page 5, Line 104…the authors have inserted a subheading for “Responders and Non-Responders”. However, after including a section “Definition of Responders” and clearly elaborating on “Responders”, the reader is left in the dark about a definition for “Non-Responders”? Therefore, following on from the questions raised in Paragraphs 1a, b, c above…One would like to suggest that the authors should clearly define who the ‘Non-responders’ in this study are, and also to indicate whether they are the same as ‘unnecessary participants’ and ‘non-informative participants…. This will assist in enhancing the clarity and comprehension of the arguments raised in the rest of the article/manuscript….
3. On page 9 Lines 178-182, the authors have added a subsection on “Ethical losses and unnecessary participants”. Here the authors state, “A source of recurring dispute within RCTs is their ethical acceptability. For an individual, participating in an RCT can mean different types of ethical loss, for example, exposure to side-effects, loss of access to standard care, or loss of access to the best available and tailored treatment.”. One would like o suggest that other important potential ethical losses which ought to be discussed would be issues of ‘moral distress and justice’…For example where individuals have volunteered for an RCT with the hope of gaining a potential benefit or even contributing to science only to find out that there their participation was perchance ‘unnecessary’…This can create some form of ‘moral distress’ and a reluctance to participate in future RCTs. Furthermore, with regards to justice, where individuals have invested time and effort or money e.g., taking time off work to participate in an RCT, it could be considered an injustice or unjust and unethical to use peoples time and effort without any justifiable benefits either for themselves or to society in general…. This would be the case regardless of whether the individual subjects participation was covered under the informed consent doctrine? …
4. On pages 17-18, Lines 333-345, The authors have described the concept of ‘equipoise in RCTs’. One would like to suggest that moving this section to the ‘Introduction’ section from the ‘Discussion’ which could serve as a background to RCT studies and provide a justification on why it would be important to identify “responders” and “non-responders” in RCTs….??
5. Insert a statement of limitations which identifies potential limitations to assumptions made in this study…For example are there any other confounding variables which could impact on the assumptions made for this study? E, g. What about participants who withdraw prematurely from an RCT... How will this impact on the percentage or proportion of ‘responders’ and ‘non-responders’…. Because the assumptions illustrated in this case are based on the principle of ‘all things being equal’…??
6. Finally, the statistical parameters and calculations for this study should be further reviewed by another qualified biostatistician

**Minor Corrections**: Few typographical and grammatical errors…

**Recommendation**: Acceptable after major revisions and further biostatistical review
